# Stress and value: the student perspective on utilizing real vs. actor patients in objective structured clinical examinations

**DOI:** 10.1186/s12909-024-05673-y

**Published:** 2024-07-15

**Authors:** Chad Vercio, Gordon Tan, Ivanna N. Maxson, Yara Matta, Bradley Cacho, Daniel Calaguas, Amy Hayton, Soo Kim

**Affiliations:** 1 Department of Pediatrics, Loma Linda University Children’s Hospital, Loma Linda University School of Medicine, 11234 Anderson St, Loma Linda, CA 92354 USA; 2 Present Address: Department of Pediatrics, UCLA, UCLA School of Medicine, Los Angeles, CA USA; 3 Present Address: Palomar Health Medical Group- Graybill, Internal Medicine and Pediatrics, Murrieta, CA USA; 4 Department of Internal Medicine, Loma Linda University Medical Center, Loma Linda University School of Medicine, Loma Linda, CA USA; 5 Loma Linda University Children’s Hospital, Los Angeles, CA USA; 6 Loma Linda University Medical Center and Loma Linda Children’s Hospital, Murrieta, CA USA

## Abstract

**Background:**

Studies have shown objective structured clinical examinations (OSCEs) to be one of the most reliable tools in assessing clinical performance; however in Pediatrics they primarily use manikins, simulators or parent actors which limits the comprehensiveness of the assessment. In our Pediatric Clerkship, medical students are evaluated using a standardized rubric during a clinical evaluation exercise (CEX) with real patients. This study assessed medical students’ perceived stress levels and the educational value of the CEX compared an OSCE. We hypothesized there would be equal stress and value for students with the CEX experience compared to the OSCE.

**Methods:**

Third year students anonymously completed questionnaires after required Pediatric CEX and Internal Medicine OSCE evaluations from July 2016-June 2017. The questionnaire included questions from the Intrinsic Motivation Inventory, a validated tool used to assess feelings of stress and perceived value of an exercise.

**Results:**

A total of 147 and 145 questionnaires were completed after the CEX and OSCE. There were no differences between groups regarding levels of “nervousness” (*p* = 0.543) and “relaxation” (*p* = 0.055); students felt more “at ease” (*p* = 0.002) and less “pressure” (*p* < 0.001) during the CEX. Students perceived the CEX to be more useful and important to improve skills compared to the OSCE for the history taking, physical exam and interpersonal skills.

**Conclusions:**

Our results indicate that the CEX was associated with lower stress levels and had higher perceived value when compared to the OSCE. This study supports the usefulness of incorporating real patients into the clinical evaluation of medical students.

## Background

Since Dr. Ronald Harden’s inception of the objective structured clinical examination (OSCE) in the 1970’s the OSCE has spread around the globe and cemented itself as an educational pillar spanning all levels of training and a wide range of health professions [1–6]. Today, OSCE’s serve both summative and formative functions [7–9]. They offer the ability to have nearly identical encounters which increased the possibility of reliable, valid and replicable assessments with definable goals for performance which allows for the use of OSCEs as a summative assessment of a trainee. This method reduces biases and variability associated with other assessment methods, and filling in the gaps missed by assessment methods focused primarily on knowledge [10–12]. There is also value in OSCEs for formative purposes as they create a safe clinical learning experience with feedback, ideally immediate, given in a way that contributes to growth and preparation for future clinical encounters and exams. There is evidence that formative focused OSCE experiences may also improve performance on summative clinical assessments [13–15]. However unfortunately the opportunity for formative feedback is missed as frequently this is not performed.

OSCEs typically use standardized patients (SPs), which were first introduced in 1964, for clinical based encounters and provide similar clinical encounters for each student.^13^ Studies have shown OSCEs to be the most reliable and accurate tool in assessing performance and clinical acumen compared to other methods including modules, simulations, and case discussions [10, 14]. The specific components and modifications of the OSCE vary between institutions, but the vast majority include SPs and/or computerized simulations.

Significant research has been done to evaluate OSCE’s summative qualities, connecting OSCE scores to performance on other OSCEs, knowledge based exams, clinical evaluations and clerkship performance, national licensing examinations, and performance in residency [15, 16]. The OSCE has been implemented and studied in numerous formats, including at the international level in many countries for medical licensing processes [17, 18]. Additionally OSCE’s serve a purpose in meeting the societal expectations for the evaluative rigor of clinical training.

As the OSCE gained importance in medical education, the use of OSCEs with direct “patient” contact in the field of Pediatrics lagged behind due to the innate difficulty of standardizing young children and infants as SPs. The challenges of having standardized actor pediatric patients range from performance fatigue requiring a large volume of pediatric SPs, standardization of these performances, and the potential psychological effects on the pediatric SPs, particularly in youngest patients. These patients are unlikely to completely understand what is occurring and this may disrupt the relationship with their own primary care provider [19–21]. Although some groups have invested significant time and resources into developing pediatric actor SP OSCEs, these have not become widespread in medical education [22, 23]. These challenges have caused Pediatric Clerkships to avoid interaction with a standardized patient for an OSCE although there is more recent literature that has demonstrated feasibility [24, 25]. 

There are additional methods to assess clinical performance of trainees in clinical settings including the Clinical Evaluation Exercise (CEX) and mini-CEX. These have been studied for adult and pediatric populations and determined to varying degrees to be reliable and valid summative and formative assessment tools [26–28]. The mini-CEX is now favored and deployed in many residency training programs as part of the method of assessing trainee competence and provide formative feedback for trainees. It is promoted by the American Board of Internal Medicine (ABIM), which encourages its use on every clinical rotation. It has been shown to have superior reproducibility to the traditional CEX and is more easily implementable due to the increased efficiency in assessment of a trainee.[29] There are a few studies in the literature which show feasibility and positive impact on clinical skills in medical students [30, 31]. 

The Pediatric Clerkship at Loma Linda University School of Medicine (LLUSM) has a long history of utilizing a real, non-actor, non-standardized patient encounter as part of medical student training and evaluation. This study assessed perceived stress levels and the perceived educational value of the non-actor clinical examination exercise (CEX) compared to a traditional OSCE from the student perspective. We are not aware of any previous literature that has assessed the learner perception of value or stressfulness of the activity between a CEX or OSCE. It has been described in the literature that clinical experiences such as OSCEs are stressful for students; however, they do recognize their utility for formative and summative evaluations [32–34]. We hypothesized that there would be no difference in stress or value between the two different summative evaluation encounters.

## Methods

A cross-sectional study was performed to evaluate medical student perceptions comparing the real patient CEX to the standardized patient actor OSCE. This study received Institutional Review Board (IRB) exemption given the minimal risk to participants and anonymous data collection. Students were asked about the perceived value of the CEX and OSCE for improving clinical skills, in addition to stress levels experienced during each exam. Eligible study participants consisted of all third-year medical students at LLUSM rotating through the required Pediatric and Internal Medicine (IM) clinical clerkships during the 2016–2017 academic year (*n* = 165).

Study participation was voluntary, and all participating students were verbally consented to complete anonymous questionnaires related to their perceptions of the clinical and learning assessment experiences for the purpose of medical education research. It was emphasized to students that there was no penalty for students who did not participate. Questionnaires were administered immediately after routine Pediatric CEX and IM simulated patient OSCEs and prior to receiving their grades for the OSCE or CEX. At LLUSM, third year students are assigned to rotation sequences for the required clerkships of Family Medicine, Obstetrics and Gynecology, Internal Medicine, Neurology, Psychiatry, Surgery, and Pediatrics. The sequence of clerkships follows the same progression, with the order of rotation determined by the initial clerkship. In this scheduling format, approximately 60% of participating students took the Pediatric CEX prior to the IM OSCE, and 40% of participating students vice-versa. On the Pediatric rotation the CEX took place during the outpatient portion of the clerkship and could be scheduled between weeks 2–7 of the 8 week clerkship. The IM OSCE took place in the last week of the 10 week clerkship. Students participate in OSCEs in the pre-clinical years and have OSCEs in some form on every other rotation throughout the year.

The CEX was administered using a non-actor, non-standardized patient at the pediatric resident clinic, both at a hospital-based clinic in Loma Linda, California for one month of the study period and at a federally qualified health center in San Bernardino, California throughout the rest of the study period. The two different locations were due to a scheduled move of the pediatric resident clinic. There were 4 faculty involved in the direct observation of students and delivery of feedback. The students had regular interactions with the faculty administering the CEX as they were involved in delivering didactic sessions with the students. The CEX evaluator did not interject during the student’s portion of the encounter and allowed the student to complete their history, exam, assessment and plan with the patient and family. The faculty was ultimately responsible for the medical care of the patient and the student did have the opportunity to observe the interaction between the faculty and patient/family as part of their CEX. This clinic is also a resident run clinic and often the CEX encounters are actually shorter than the resident based encounters. Due to the focus on education both for medical students and residents the administration had no concerns about assessment methods within the clinic.

Potential CEX patients were screened by pediatric faculty CEX evaluators to include well child appointments or simple acute visits without serious chronic ongoing medical problems such as: fever, rash, acute otitis media, upper respiratory infection symptoms, constipation, gastroenteritis, feeding issues, abdominal pain, or reflux symptoms. All CEX patients were 5 years of age or younger (included newborn visits) and had an English-speaking family. Participating caregivers were informed their child appeared to be appropriate for an assessment of a student and provided verbal informed consent to participate in the CEX. Medical students were provided the name, sex, and age of the patient less than 5 min prior to the encounter. They were not informed of the reason for the visit. Each student was allotted 30 min to complete the encounter, which included obtaining the chief complaint, history, physical exam, and formulating an assessment and plan with appropriate parental counseling. Each encounter was observed in the room by a member of a core group of Pediatric Clerkship faculty who received training on CEX encounter assessment.

The IM OSCE was completed in the clinical skills center, which is utilized for other LLUSM OSCEs throughout all 4 years of medical training. Adult standardized patient actors were utilized who had received 2–3 h of OSCE training. These actors were obtained, recruited, and trained by the clinical skills center at LLUSM. Students completed two clinical encounters: one 15-minute encounter and a second 20-minute encounter during which a point-of-care ultrasound examination was performed. Both encounters required completion of a history, physical exam, assessment and plan, patient counseling, and the IM OSCE involved documentation of a note. Students were told only the patient chief complaints prior to the exam and were provided a prompt with information on the age, sex, vitals, and more detailed chief complaint with some context immediately prior to each encounter. Chief complaints for the IM simulated patient encounters were abdominal pain and shortness of breath. Internal Medicine OSCEs were observed directly through a two-way mirror, graded in-real-time, and were recorded for future student review. Four Internal Medicine faculty members evaluated the encounter using a standardized rubric, which evaluated dimensions of the history, appropriate systems for physical exam, differential diagnosis, appropriate work-up, and quality of note (including differential diagnosis support). After completing each encounter, students were given 10 min to write a clinical note documenting the history, physical exam, differential diagnosis with supporting evidence, and plan, which also contributed to the overall OSCE grade.

The dimensions assessed in both the Pediatric CEX and Internal Medicine OSCE were similar with a rubric that was the basis for feedback. They included evaluation of the comprehensiveness of the history of present illness, physical exam, differential diagnosis/assessment, plan, and information sharing. Because the IM OSCE involved standardized patients with specific chief complaints, there were more specific history dimensions that were relevant to the chief complaint on which students were evaluated.

After both the Pediatric CEX and Internal Medicine OSCE, an individual feedback session was provided by the grading faculty member. Following this feedback, students were offered the opportunity to complete the research questionnaire. There were four Internal Medicine faculty performing the CEX and OSCE and providing feedback in each clerkship. The CEX and OSCE were both moderately important as they were necessary to pass the clerkship and factored significantly into grades.

The questionnaire consisted of 14 survey questions derived from the “Pressure/Tension” and “Value/Usefulness” statements of the Intrinsic Motivation Inventory, a validated tool used to assess feelings of stress and perceived value of an exercise, hereafter referred to simply as concepts of “stress” and “value.” [35]. Each question was answered using a five-point Likert agreement scale rating perceptions related to concepts of “value” and “stress.” Subgroups of the 14 questions were analyzed according to 11 concept categories: “real life clinical scenario,” feelings of “nervousness,” “relaxed,” “pressured,” and “at ease,” “usefulness” and “importance” for improving clinical skills, accuracy for assessing clinical skills. Median scores and interquartile ranges were calculated for each survey item. Scores for each of the 14 questions and each of the concept categories were compared across the two groups. The Likert scale responses were numbered 1–5 and a Kruskal-Wallis and Mann Whitney U tests were used to test for differences between the various scores (reported as medians) in the CEX and OSCE groups. We looked for differences in distribution between the groups using Kolmogorov-Smirnoff. Statistics were performed with SPSS v 28 (SPSS, IBM).

In addition to the Likert-scaled questions, students completed two free-response short answer prompt questions: (1) What factors contributed to the amount of nervousness or pressure you felt during this OSCE?; (2) What was the most helpful aspect of this OSCE? For the free response questions, answers were coded by two investigators (SK and YM), grouped by theme, and tallied according to frequency of theme occurrence.

## Results

Out of 165 third year students who completed Pediatric and IM clerkships, 147 (89%) and 145 (88%) completed CEX and OSCE questionnaires, respectively. Scores for various questions assessing stress levels during the CEX and OSCE as well as their perceived educational value were compared across the two groups.

**Fig. 1:**
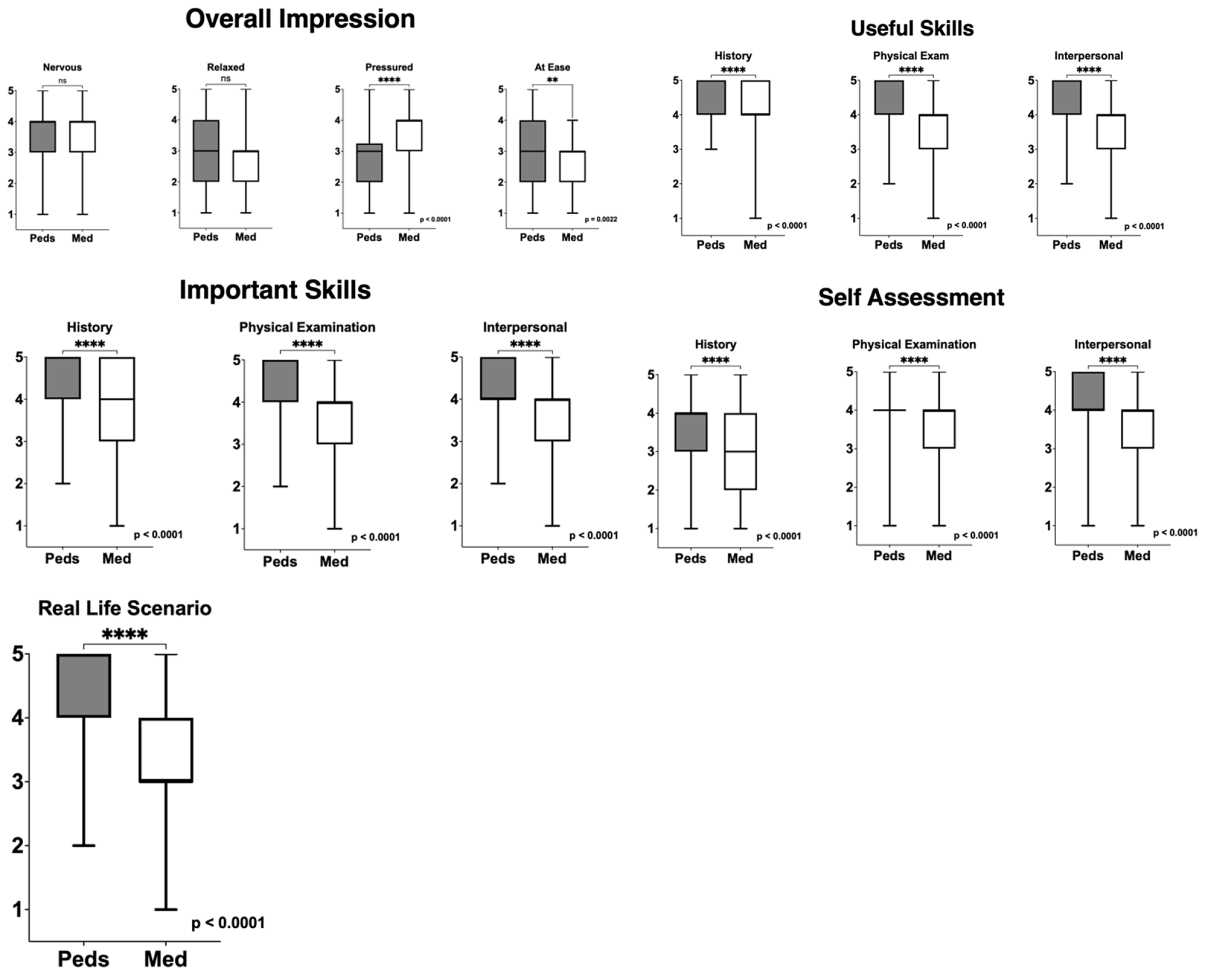
Comparison of Internal Medicine OSCE and Pediatric CEX Scores

Results showed a significant difference across the two groups in feeling pressure during the scenario with median scores lower in the CEX compared to the OSCE group (Median CEX Score 3, Median OSCE Score 4, *p* < 0.001) which is shown in both Table 1; Fig. 1. Students also reported feeling more at ease during the CEX than during the OSCE (Median CEX Score 3, Mean CEX Score 3.16, Median OSCE Score 3; Mean OSCE Score 2.81; p 0.002). There was no significant difference between the two groups in feeling nervous (Median CEX Score 4; Median OSCE Score 4; *p* = 0.543) or relaxed (Median CEX Score 3; Median OSCE Score 3; *p* = 0.055) during the encounter.

Median scores were calculated for how important and useful the encounters were at improving each skill set (history-taking, physical exam, and interpersonal communication) and for how accurately the encounters represented those skill sets. There was a significant difference in median scores showing that medical students found the CEX to be more useful (Median CEX Score 5 and Median OSCE Score 4, *p* < 0.0001) and important than the OSCE (Median CEX Score 5 Median OSCE Score 4; *p* < 0.0001) in improving their history-taking, physical exam, and their interpersonal skills. Additionally, scores also showed that students perceived the CEX more accurately represented their skills compared to the OSCE (Median CEX Score 4, Median OSCE Score 4; Mean CEX score 3.874; Mean OSCE Score 3.375, *p* < 0.0001).

Overall, there was a significant difference between the two groups, with students showing a greater perception of improvement in, but also accurate representation of, their history-taking skills (Median CEX Score 4, Median OSCE Score 3, *p* < 0.001), physical exam skills (Median CEX Score 4, Mean CEX Score 3.91, Median OSCE Score 4, Mean OSCE Score 3.4, *p* < 0.001), and interpersonal skills (Median CEX Score 4, Mean CEX score 4.1, Median OSCE Score 4, Mean OSCE Score 3.59, *p* < 0.001). Finally, results also showed that students perceived the CEX to more accurately represent a real-life clinical scenario (Median CEX Score 5, Median OSCE Score 3, *p* < 0.0001) than the OSCE. (Table 2)

The results from the first free response questions are noted in Table 2. Factors contributing to the degree of nervousness or pressure during the encounters showed that having the attending physically in the room during the CEX was mentioned most (44 comments), followed by the knowledge that it was a graded encounter (40 comments), uncertainty about the patient’s history and chief complaint (29 comments), and simply being observed (24 comments). Students in the OSCE reported that the timed nature of the encounter most contributed to feeling pressured (62 comments), followed by knowledge of the graded encounter (45 comments), the feeling of “being watched” (21 comments), and concern and uncertainty over missing key details (17 comments).

## Discussion

Our data show that the students rated their performance during the CEX as a more accurate representation of their day-to-day interactions with patients and more valuable to their growth as a physician. Students felt it was more useful and more important for the improvement of their history taking, physical exam and interpersonal skills than with a standardized patient. The data supported our hypothesis that the real patient encounter was viewed as more valuable than an OSCE encounter. The increase in recognized value could be related to a removal of the “theatrics” related to OSCEs. This has been described with OSCEs as an underlying theme of disingenuity and insincerity due to the theatric nature where students put on a performance they believe will move the audience (the evaluators) [36]. Evaluating medical students during real-patient encounters, such as the CEX, may therefore reveal a more accurate window of true clinical competency with a reduction in theatrical performance.

There were several distinct factors that we would have expected to increase stress with the CEX on the Pediatric Clerkship rotation. It was performed with the evaluating physician in the same room as the trainee, which was noted in the qualitative portion to be a very commonly mentioned theme around stress in the encounters. Additionally, for the vast majority of students, the CEX was performed in an unfamiliar clinical environment they had not encountered previously. Despite these factors, which may have increased stress or anxiety, the CEX was found to be no more stressful or anxiety-provoking than the standardized OSCE encounters.

While our methods involved a full CEX with both formative and summative purposes, it is reasonable to extrapolate to a mini-CEX, which could be used for solely formative purposes and would likely decrease the level of stress or anxiety a trainee experiences compared to a standard CEX or OSCE. This would allow medical students to receive feedback in the moment on direct patient encounters, and faculty should be reassured that it may be viewed as extremely valuable and not any more stressful than a standardized OSCE encounter.

This study was inherently limited by only having included a single institution and was an asymmetrical comparison of two separate clinical evaluations in two different clinical fields and patient populations: Internal Medicine and Pediatrics. Students were rating two different educational experiences, and it is possible they viewed the CEX as a complete unit decreasing their rating of the overall stress associated with the observed portion of the encounter. It is also possible that the nature of working with children resulted in a less stressful environment for students. Without the ability to control for other factors, such as the inherent differences in the clinical fields and patients, and clerkship experiences such as directors, teaching and evaluating faculty, residents, and clinical clerkship duties, there was clear risk for confounding bias.

This study overall supports the usefulness of incorporating a real patient into the evaluation of medical students during their medical school clerkships. This is the first study to our knowledge comparing self-reported stress levels and perceived value of a real patient encounter as in the CEX to a standardized actor OSCE. Further research should be performed to evaluate the utility of this method in medical education and how it translates into actual learning.

One of the primary questions relating to a CEX in any setting is the sustainability of the assessment. Ensuring that faculty are compensated for this time is critical to maintain their interest in contributing to students’ development. This assessment was incorporated into the time the clerkship physician leaders were allocated for clerkship responsibilities and supported by the Pediatric Department. With medical schools facing challenges in having directly observed encounters for students, supporting Pediatric clerkships to perform this and provide both formative and summative feedback experiences would be highly valuable [37, 38]. 

## Conclusions

Our data show that students perceived their performance during the CEX as more accurately representative of their day-to-day interactions with patients. In addition, the real patient encounter was rated by medical students as more useful and more important for improving their history taking, physical exam, and interpersonal skills than evaluating a standardized patient. We believe that this provides clerkship directors with appropriate reasoning to incorporate a CEX into their evaluation of students on their clerkship.

Table 1 Median (interquartile range) Likert agreement scale scores for Intrinsic Motivation Inventory questions on students’ perceptions of “stress” and “value” in clinical examinations. Likert scale responses were as follows: 1-strongly disagree, 2-disagree, 3-neutral, 4-agree, 5-strongly agree. Scores from the Internal Medicine OSCE were compared to the Pediatric CEX using Kruskal-Wallis testing, with *p* ≤ 0.05 considered statistically significant.

**Table 1:** Median intrinsic motivation inventory “stress” and “value” scores for Internal Medicine OSCE and Pediatric CEX examinations

Characteristics	Internal Medicine OSCE (*n* = 145)	Pediatric CEX (*n* = 147)	*p* value
The exam represented a real-life clinical scenario	3 (2–4)	5 (4–5)	< 0.0001
I felt…			
Nervous	4 (3–5)	4 (3–5)	0.543
Relaxed	3 (2–4)	3 (1–5)	0.055
Pressured	4 (3–5)	3 (2–4)	< 0.001
At ease	3 (2–4)	3 (1–5)	0.002
The exam was useful for improving			
History taking skills	4 (3–5)	5 (4–5)	< 0.001
Physical exam skills	4 (3–5)	5 (4–5)	< 0.001
Interpersonal skills	4 (3–5)	5 (4–5)	< 0.001
The exam was important in improving			
History taking skills	4 (3–5)	5 (4–5)	< 0.001
Physical exam skills	4 (3–5)	5 (4–5)	< 0.001
Interpersonal skills	4 (3–5)	4 (3–5)	< 0.001
My performance accurately represented my			
History taking skills	3 (1–5)	4 (3–5)	< 0.001
Physical exam skills	4 (3–5)	4 (IQR 0)	< 0.001
Interpersonal skills	4 (3–5)	4 (3–5)	< 0.001

Table 2 Themes extracted from two free response questions asked immediately after completing Internal Medicine OSCE and Pediatric CEX examinations. The frequency count of each theme is given in addition to the percentage out of total responses. The first question asked about factors contributing to feeling nervousness or pressure during the exam. The second question asked for perceptions on the most helpful aspect of the exam. Response themes were generally similar between Internal Medicine and Pediatric examinations.

**Table 2:** Themes from free response questions on factors contributing to perceptions of “stress” and “value” in the Internal Medicine OSCE and Pediatric CEX exams

Internal Medicine OSCE (*n* = 145)		Pediatric CEX (*n* = 147)	
Theme	No. responses (%)	Theme	No. responses (%)
Factors contributing to feeling nervousness or pressure?			
“Time”	62 (42.8)	“Attending in room”	44 (29.9)
“Graded”	45 (31.0)	“Grading”	40 (27.2)
“Being watched”	21 (14.5)	“Uncertainty”	29 (19.7)
“Uncertainty/ missing things”	17 (11.7)	“Observed”	24 (16.3)
Most helpful aspect of this exam?			
“Feedback”	43 (29.7)	“Immediate feedback”	87 (59.2)
“Step 2 preparation”	17 (11.7)	“Authenticity of real patient”	49 (33.3)
“Time”	15 (10.3)	“More comfortable with real patient”	13 (8.8)
“Realistic patients”	8 (5.5)		

## Data Availability

I have uploaded the original data as a related file. The datasets generated and/or analysed during the current study are not publicly available but are available from the corresponding author on reasonable request.
